# Correlates for cardiovascular diseases among diabetic/hypertensive patients attending outreach clinics in two Nairobi slums, Kenya

**DOI:** 10.11604/pamj.2014.19.261.5261

**Published:** 2014-11-10

**Authors:** Gladys Mugure, Mohamed Karama, Catherine Kyobutungi, Simon Karanja

**Affiliations:** 1 Jomo Kenyatta University of Agriculture and Technology-College of Health Sciences, Nairobi, Kenya; 2 Kenya Medical Research Institute, Mbagathi Way, Nairobi City, Kenya; 3 African Population and Health Research Centre, Nairobi, Kenya

**Keywords:** Cardiovascular diseases, diabetes, hypertension, Kenya, slums

## Abstract

**Introduction:**

Cardiovascular diseases (CVD) are the leading cause of death in the world. Over 80% of CVD related deaths occur in low- and middle-income countries (LMICs). Diabetes and hypertension, whose prevalence in Kenya is on the rise, are major risk factors for CVD. Despite this, studies indicate that awareness on the management of risk factors for CVD among diabetic/hypertensive patients in African populations is generally low. The aim of the study was to determine the risk factors for CVD among diabetic and/or hypertensive patients attending diabetes and hypertension management clinics in Korogocho and Viwandani slums of Nairobi.

**Methods:**

Data were collected using questionnaires administered to 206 diabetic/hypertensive patients attending the clinics between July 2010 and February 2011. A review of these patients’ medical records was done to determine the history of CVD outcomes such as hypertensive heart diseases, stroke and peripheral arterial diseases.

**Results:**

Majority (66.5%) of the study participants were females mainly in the 51-65 age category. The study findings revealed that 73 (33.4%) respondents had CVD outcomes. In addition, 41.8% of the respondents were not aware of the causes of diabetes/hypertension. Age category 51-65 years had the highest (43.8%) number of respondents with CVD. Sex of the respondents and awareness of the link between hypertension and CVD were significantly associated with CVD outcomes (p<0.05) among the respondents.

**Conclusion:**

Measures to improve awareness levels among patients at high risk of CVD outcomes are needed to complement other measures to reduce CVD risk among such patients.

## Introduction

Despite the already existing burden of communicable diseases in low- and middle-income countries (LMICs), non-communicable diseases (NCDs) are increasingly a burden in LMICs affecting people in the prime of their lives and putting more pressure on already stretched health systems, governments and family budgets. Non-communicable diseases disproportionately affect LMICs where 80% of all deaths occur; cardiovascular diseases account for most NCDs deaths [1]. Worldwide, cardiovascular diseases (CVD) are the leading cause of death and a major cause of disability and lost productivity in adults [2]. Deaths from NCDs occur at earlier ages in LMICs compared to high income countries. The World Health Organization (WHO) estimated that 17.3 million people died from CVD in 2008, representing 30% of all deaths globally [3]. Of these deaths, an estimated 7.3 million were due to coronary heart disease and 6.2 million were due to stroke [3]. Indeed, CVD is the leading cause of morbidity and mortality in LMICs [4] and in sub Saharan Africa [5] in spite of infectious diseases such as malaria, HIV/AIDS and tuberculosis which continue to pose major health risks. There is thus need to control CVD as they undermine health, shorten life expectancy, and cause enormous suffering, disability, and economic costs [6].

The increase of the CVD burden in LMICs is a result of an increase in the prevalence of risk factors and lack of access to preventive interventions [7]. Hypertension is the leading risk factor for CVD worldwide. Globally, nearly one billion people have hypertension, where two thirds are in LMICs. In sub-Saharan Africa (SSA), hypertension remains the most threatening risk factor, with national prevalence ranging between 15% and 30% in adults [8]. Diabetes is also a major risk factor for CVD and about 60% of all mortality in people with diabetes results from CVD events. Cardiovascular risk increases with raised glucose values [9] and CVD is the largest contributor to the direct and indirect costs of diabetes mellitus [10].

Chronic conditions such as hypertension and diabetes require patient education to achieve adequate control and prevent adverse health outcomes including CVD outcomes. Patients with hypertension may need to understand how to take their medications properly and modify their lifestyle to achieve adequate blood pressure control [11]. The intricacies of the diabetic diet, treatment with insulin injections, and home glucose level monitoring, place educational requirements on patients [12]. Patient education plays a critical role in facilitating patients′ acceptance of their diagnosis and understanding changes required for active participation in management of their condition [13]. The importance of health education to increase awareness in the community on the preventive aspects of chronic conditions including diabetes and hypertension cannot be overemphasized [14]. From focus group discussions conducted in the United States, participants with diabetes demonstrated a significant lack of awareness of the link between diabetes and CVD events [15].

Studies done among residents in the two Nairobi slums have shown low awareness levels and poor control of diabetes and hypertension [16, 17]. There is no doubt that there is a need for effective CVD prevention programs but for these programs to be effective and successful, it is important to establish the CVD risk factors in the intended beneficiary population. This need is even more critical in LMICs settings that are grappling with multiple disease burdens and poorly functioning health systems. This study was therefore carried out to determine the risk factors for CVD among diabetic/hypertensive patients attending diabetes and hypertension management clinics in two Nairobi slums.

## Methods

### Study site

The study was carried out at two outreach diabetes and hypertension management clinics in Korogocho and Viwandani slums, Nairobi County. The two slums are located about 5-10 km from the Nairobi Central Business District and have government public health facilities serving their communities. The clinics were selected purposively to capitalize on data collection efforts during an on-going project aimed at providing access to high quality care in the management of the two conditions for residents of the two slums.

### Study design and sampling

This was a descriptive cross sectional study involving 206 patients selected using systematic random sampling procedure. The first patient was selected randomly after which every 5th patient who visited the clinic was selected. Patients were sampled from the two study sites until the required sample size was achieved. The study participants included those 18 years and above who were willing to participate and give written informed consent.

### Data collection

Semi-structured questionnaires were administered to the respondents to seek information on the socio-demographic characteristics, behavioural factors, patients’ awareness on the cause of diabetes/hypertension and the management of the two conditions. In addition, participant's medical records were analyzed to determine those with a history of CVD outcomes/events. The CVD studied included coronary heart diseases, cerebrovascular diseases and peripheral arterial diseases.

### Analysis

STATA version 12.0 was used for analysis. Descriptive statistics were used to describe the measures of central tendency and spread. Chi-square test of independence was used to estimate the association of socio-demographic factors, behavioural factors and patients’ awareness on diabetes and hypertension factors with regard to CVD. Multiple logistic regressions were fitted to assess the associations of the different factors with CVD. Estimates with p<0.05 were deemed statistically significant.

### Ethical approval

Scientific and ethical approval to conduct the study was obtained from National Ethical Review Committee of the Kenya Medical Research Institute. Data collected from study participants was kept confidential.

## Results

### Characteristics of the study participants

The mean age and the standard deviation of the study participants were 55.4±(0.85) years and majority were females. The prevalence of CVD outcomes among the study participants was 33.4%. Majority (51.0%) of respondents were in the age category 51-65 years. Half of the respondents were self-employed and most (58.3%) of the respondents had at least acquired primary school level of education. The other characteristics are presented in Table 1.

**Table 1:** Characteristics of diabetic/hypertensive patients attending diabetes and hypertension management clinics in two Nairobi slums

Variable	Frequency	%	95% CI
**Sex**			
Male	69	33.5	27.1-40.4
Female	137	66.5	59.6-72.9
**Age groups**			
18-35 years	11	5.3	2.7-9.4
36-50 years	50	24.3	18.6-30.7
51-65 years	105	51.0	43.9-58.0
> 66 years	39	18.9	13.8-25.0
**Marital status**			
Single	25	12.1	8.0-17.4
Married	136	66.0	59.1-72.5
Divorced/separated	13	6.3	3.4-10.5
Widow/Widower	31	15.1	10.5-20.7
**Levels of education**			
No education	43	20.9	15.5-27.1
Primary education	120	58.3	51.2-65.1
Secondary education	43	20.9	15.5-27.1
**Occupation**			
Self-employed	103	50.0	50.0-57.0
Temporary employment	24	11.7	7.6-16.8
Permanent employment	12	5.8	3.0-10.0
Farmer	19	9.2	5.6-14.0
Not employed	48	23.3	17.7-29.7
**Clinic attendance**			
As required	185	89.8	84.8-93.6
When not feeling well	3	1.5	0.3-4.2
In need of more medicine	5	2.4	0.8-5.6
In need of medical check ups	11	5.3	2.7-9.3
**Cause of diabetes/hypertension**			
Hereditary	30	14.6	10.0-0.1
Lifestyle disease	72	35.0	28.5-41.9
Didn't know	86	41.8	34.9-48.8
Others e.g. fainting	16	7.8	4.5-12.3
**Can diabetes/hypertension be prevented?**			
Yes	170	82.5	76.6-87.4
No	10	4.9	2.3-8.7
Not sure	26	12.6	8.4-18

### Awareness on diabetes and hypertension factors

Majority (96.8%) of the diabetic respondents were aware that it was important to take medication as required. This was also observed among the hypertensive respondents where 92.8% of the respondents reported that it was important to take medication as required. Majority (78.3%) of the hypertensive patients reported they were taking medication to regulate their blood pressure. Most (47.1%) of the hypertensive respondents were not sure of the link between diabetes and CVD (Table 2).

**Table 2:** Awareness on diabetes and hypertension factors among diabetic and/or hypertensive patients attending diabetes and hypertension management clinics in two Nairobi slums

Variable	Frequency	%	95% CI
**Diabetic respondents**			
**Is it important to take medication as required?**			
Yes	153	96.8	92.8-99.0
No	5	3.2	1.0-7.2
**Reasons for taking medication**			
To satisfy doctors requirement	22	13.9	8.9-20.3
To regulate blood sugar	125	79.1	71.9-85.2
Others e.g. to get well	11	7.0	3.5-12.1
**Consequences of interrupted treatment**			
Rise in blood sugar	96	60.8	52.7-68.4
Drug resistance	10	6.3	3.1-11.3
Fatality	19	12.0	7.4-18.1
Don't know	33	20.9	14.8-28.1
**Is there a link between diabetes and CVD?**			
Yes	52	32.9	25.7-40.8
No	80	50.6	42.6-58.7
Don't know	26	16.5	11.0-23.2
**Hypertensive respondents**			
**Is it important to take medication as required?**			
Yes	128	92.8	87.1-96.5
No	10	7.3	3.5-12.9
**Reasons for taking medication**			
To satisfy doctors requirement	18	13.0	7.9-19.8
To regulate blood pressure	108	78.3	70.4-84.8
Others e.g. to get well	11	8.0	4.0-13.8
**Consequences of interrupted treatment**			
Rise in blood pressure	95	68.8	60.4-76.4
Drug resistance	5	3.6	1.2-8.3
Fatality	12	8.7	4.6-14.7
Don't know	26	18.8	12.7-26.4
**Is there a link between hypertension and CVD?**			
Yes	53	38.4	30.3-47.1
No	65	47.1	38.6-55.8
Don't know	20	14.5	9.1-21.5

### Behavioral factors among the respondents

About 28.2% of the respondents had ever consumed alcohol; in addition, 4.4% of the respondents were currently consuming alcohol. Majority (77.7%) of the respondents were taking food containing salt. A summary of the behavioural factors are presented in Table 3.

**Table 3:** Behavioural factors of diabetic/hypertensive patients attending diabetes and hypertension management clinics in two Nairobi slums

Variable	Frequency	%	95% CI
**Salt intake**			
Adds salt to food	21	10.2	6.4-15.2
Takes salted food	160	77.7	71.4-83.2
Does not use salt	25	12.1	8.0-17.4
**Sugar consumption**			
Yes	37	18.0	13.0-23.9
No	169	82.0	76.1-87.0
**Soft drinks intake**			
Yes	34	16.5	11.7-22.2
No	172	83.5	77.7-88.3
**Smoking status**			
Never smoked	169	82.0	76.1-87
Current smoker	4	1.9	0.5-4.8
Former smoker	33	16.0	11.3-21.8
**Tobacco products usage**			
Never used	193	93.7	89.5-96.6
Currently using	5	2.4	1.7-7.5
Currently not using	8	3.9	0.8-5.6
**Alcohol intake**			
Never a drinker	139	67.5	60.6-73.8
Current drinker	9	4.4	2.0-8.1
Former drinker	58	28.2	22.1-34.8

### Prevalence of CVD with regard to characteristics of diabetic/hypertensive patients

Males had the highest (47.8%) occurrence of CVD outcomes compared to females (29.2%). Respondents in the age category 51-65 years had the highest (43.8%) occurrence of CVD outcomes compared to the other age categories. About 44.2% of the respondents with CVD had not acquired any formal education. At least 38.4% of the respondents with CVD were not aware of the cause of diabetes and/or hypertension. In addition, 38.5% of the respondents with CVD were not sure that diabetes/hypertension is preventable (Table 4).

**Table 4:** CVD prevalence in relation to characteristics of diabetic/hypertensive patients attending diabetes and hypertension management clinics in two Nairobi slums

		CVD				95% CI		
Variable	Yes	%	No	%	OR	Upper	Lower	p value
**Sex**								
Male	33	47.8	36	52.2	Ref			
Female	40	29.2	97	70.8	2.22	1.22	4.05	0.009
**Age groups**								
18-35 years	2	18.2	9	81.8	Ref			
36-50 years	12	24.0	38	76.0	1.14	0.27	7.50	0.679
51-65 years	46	43.8	59	56.2	3.51	0.72	17.03	0.119
> 66 years	13	33.3	26	66.7	2.25	0.42	11.96	0.341
**Marital status**								
Married	52	38.2	84	61.7	Ref			
Single	5	20.0	20	80.0	0.40	0.14	1.14	0.087
Divorced/separated	6	46.2	7	53.9	1.38	0.44	4.35	0.577
Widow/Widower	10	32.3	21	67.7	0.77	0.33	1.75	0.535
**Levels of education**								
Primary education	40	33.3	80	66.7	Ref			
No education	19	44.2	24	55.8	1.58	0.78	3.23	0.206
Secondary education	14	32.6	29	67.4	0.97	0.46	2.03	0.926
**Occupation**								
Self-employed	38	36.9	65	63.1	Ref			
Temporary employment	4	16.7	20	83.3	0.34	0.11	1.08	0.067
Permanent employment	4	33.3	8	66.7	0.86	0.24	3.03	0.809
Farmer	6	31.6	13	68.2	0.79	0.28	2.25	0.658
Not employed	21	43.8	27	56.3	1.33	0.66	2.67	0.422
**Cause of diabetes/hypertension**								
Didn't know	33	38.4	53	61.6	Ref			
Hereditary	9	30.0	21	70.0	0.69	0.28	1.68	0.413
Lifestyle disease	24	33.3	48	66.7	0.80	0.42	1.55	0.512
Others e.g. fainting	6	37.5	10	62.5	0.96	0.32	2.90	0.947
**Can diabetes/hypertension be prevented?**								
Yes	61	35.9	109	64.1	Ref			
No	2	20.0	8	80.0	0.45	0.92	2.17	0.318
Not sure	10	38.5	16	61.5	1.11	0.48	2.61	0.799

### CVD prevalence in relation to diabetes and hypertension factors among the respondents

About 42.3% of the diabetic respondents with CVD were not aware of the link between diabetes and CVD. Among the hypertensive respondents, about 40.0% of the respondents with CVD reported that they were not aware of the link between hypertension and CVD (Table 5).

**Table 5:** CVD prevalence in relation to diabetes and hypertension awareness among patients attending diabetes and hypertension management clinics in two Nairobi slums

		CVD				95% CI		
Variable	Yes	%	No	%	OR	Lower	Upper	p value
**Diabetic respondents**								
**Is it important to take medication as required?**								
Yes	58	37.9	95	62.1	Ref			
No	4	80.0	1	20.0	0.41	0.45	3.75	0.430
**Reasons for taking medication**								
To regulate blood sugar	49	39.2	76	60.8	Ref			
To satisfy doctors requirement	6	27.3	16	72.7	0.58	0.21	1.59	0.290
Others e.g. to get well	4	36.4	7	63.6	0.89	0.25	3.19	0.853
**Consequences of interrupted treatment**								
Rise in blood sugar	34	35.4	62	64.6	Ref			
Drug resistance	1	10.0	9	90.0	0.20	0.25	1.67	0.138
Fatality	9	47.4	10	52.6	1.64	0.61	4.43	0.328
Don't know	15	45.5	18	54.6	1.52	0.68	3.39	0.307
**Is there a link between diabetes and CVD?**								
No	18	34.6	34	65.4	Ref			
Yes	30	37.5	50	65.5	0.88	0.43	1.83	0.736
Don't know	11	42.3	15	57.7	1.22	0.50	3.01	0.662
**Hypertensive respondents**								
**Is it important to take medication as required?**								
Yes	51	39.8	77	60.2	Ref			
No	2	20.0	8	80.0	0.38	0.08	1.85	0.230
**Reasons for taking medication**								
To regulate blood pressure	41	38.0	67	62.0				
To satisfy doctors requirement	7	39.0	11	61.1	1.04	0.37	2.90	0.940
Others e.g. to get well	5	45.6	6	54.6	1.36	0.39	4.75	0.628
**Consequences of interrupted treatment**								
Rise in blood pressure	34	35.7	61	64.2	Ref			
Drug resistance	2	40.0	3	60.0	1.20	0.19	7.51	0.849
Fatality	7	58.3	5	41.7	2.51	0.74	8.52	0.140
Don't know	10	38.5	16	61.6	1.12	0.46	2.74	0.802
**Is there a link between hypertension and CVD?**								
Yes	29	54.7	24	45.3	Ref			
No	16	24.6	49	75.4	3.70	1.69	8.09	0.001
Don't know	8	40.0	12	60.0	2.04	0.71	5.88	0.186

### Factors associated with CVD outcomes among the patients

The sex of study participants was significantly associated with CVD outcomes (p = 0.006). Females were less likely to develop a CVD (AOR = 0.22, 95% CI = 0.07-0.65, p = 0.006) compared to males. Respondents in the age category 51-65 years were more likely to have a CVD (AOR = 5.02, 95% CI = 0.29-86.53, p = 0.267) compared to the other age categories. Respondents’ awareness on the link between hypertension and CVD was also significantly associated with CVD (p = 0.000). Behavioral risk factors among the study participants were not significantly associated with CVD (p > 0.05) in adjusted models (Table 6).

**Table 6:** Logistic regression of factors associated with CVD among patients attending diabetes and hypertension management clinics in two Nairobi slums

Variable	Odds ratio	p value	95% CI
**Sex**			
Male	Ref		
Female	0.219	0.006	0.073-0.651
**Age group**			
18-35 years	Ref		
36-50 years	1.087	0.955	0.059-20.168
51-65 years	5.022	0.267	0.291-86.531
>66 years	4.275	0.340	0.216-84.568
**Marital status**			
Single	Ref		
Married	1.089	0.912	0.240-4.940
Divorced/separated	1.062	0.954	0 .138-8.155
Widower	1.3	0.774	0.217-7.79
**Level of education**			
No education	Ref		
Primary education	0.501	0.214	0.168-1.491
Above secondary education	1.009	0.990	0.223-4.577
**Is there a link between hypertension and CVD?**			
Yes	Ref		
No	0.145	0.000	0.054-0.386
Don't know	0.458	0.223	0.131-1.607
**Alcohol consumption**			
Yes	Ref		
No	1.071	0.883	0.430-2.665
**Salt consumption**			
Adding salt to food	Ref		
Takes salted food	0.944	0.941	0.208-4.284
Never had salt	0.976	0.980	0.143-6.642
**Soft drinks uptake**			
Yes	Ref		
No	1.243	0.699	0.413-3.740

## Discussion

### Key findings

This cross-sectional survey assessed the risk factors for CVD which should aid in preventing or delaying adverse health effects among diabetic and /or hypertensive patients in the two Nairobi slums. This is important as these patients are at a higher risk of developing CVD outcomes compared to the general population. From the present study, respondents with high CVD prevalence had not acquired formal education, were aged 51 years and above and reported no form of a source of income. Awareness of the link between hypertension and CVD and the sex of the respondents were significantly associated with CVD outcomes among the respondents.

### CVD risk factors awareness

Although age which is a non-modifiable risk factor was not significantly associated with CVD outcomes, the odds of developing adverse health effects was highest among respondents aged between 51-65 years. This age category had the highest number of respondents with CVD from the two Nairobi slums. These results were similar to the findings of a study carried out in Libya where most of the respondents with positive history of myocardial infarction were aged 55 years and above [18]. About 24.0% of respondents in the age category 36-50 years had CVD outcomes. Stroke and coronary heart disease occurs earlier in the lives of people from LMICs than in the high income countries [19]. Whereas CVD are known to be diseases of people after the age of 60 years from high income countries, they are present among Africans even before the age of 40. Age specific mortality rates associated with CVD are higher in younger age groups among men and women in Africa than in high income countries [20]. Majority of the study participants from the two Nairobi slums were females. This concurs with the findings of a study carried out in South Africa where majority of the study participants (76.3%) were females [21]. The patients attending the outreach clinics were predominantly females; this gender-bias in attendance might result from differential use of healthcare services between women and men. Previous studies have shown that females are better health care seekers than males [22].

The findings of the study showed that most of the respondents with CVD outcomes did not have formal education. These findings indicate that any interventions in this community have to take notice of the low literacy levels, especially if they entail awareness raising. Low literacy is likely to lead to lack of knowledge about hypertension and other cardiovascular risk factors and their complications, leading to poor recognition and control of hypertension [23]. In addition, lack of knowledge of appropriate target blood pressure has also been associated with poor control [24]. In order to improve CVD awareness, it is essential to provide health education often to patients attending the diabetes and hypertension management clinics as better knowledge has been shown to improve adherence to lifestyle changes and medication [25]. Glucose control improved more among patients with type 2 diabetes receiving diabetes education than those who did not [26]. Knowledge and awareness among patients can be improved via various means including social (e.g. family and friends), cultural (traditional handed-down knowledge), cross-cultural (through regional and international travel), institutions (health professionals, mass media) and self experiences in health and disease [27].

Most of the respondents were not aware that diabetes is linked to CVD. This is similar to the findings of a study in India where about 69% of the respondents were not aware that diabetes increases the risk of coronary heart diseases [28]. From a study done in Morocco, over 50% of the study population was not aware of the risks of diabetic foot [29]. These findings indicate that lack of awareness as well as lack of facilities for detection and monitoring of diabetes mellitus may contribute to the high prevalence of diabetic complications including CVD [30]. Despite diagnosis and treatment of diabetes, majority of the patients are observed to have uncontrolled blood sugar, hence the need for counseling and motivation for lifestyle modification so as to improve the management of diabetes. Awareness creation along with behavior change communication activities should be encouraged among patients for reduction of heart diseases [28].

Findings of this study showed that patients had poor awareness of the link between hypertension with CVD; similar to a study done in India where a significant number of participants did not believe that hypertension could lead to complications including CVD [31]. From the study done in India, awareness and knowledge about hypertension and its consequences were inadequate despite being perceived as serious health problems [31]. Respondents who were not aware of the cause of diabetes/hypertension had the highest occurrence of CVD. Lack of information by patients about the disease nature, causes, clinical manifestations, and management may lead to low compliance, as well as increase the incidence of complications including CVD [32]. In contrast to other studies [33, 34], behavioral factors including cigarette smoking, alcohol consumption and excessive use of salt were not significantly associated with CVD from the findings of this study. This could be due to the low prevalence of these behavioral risk factors among the study participants. This being a cross-sectional study, it is also possible that some patients had already been counselled about these risk factors and had changed their behavior.

### Study strengths and limitations

Some limitations from this study were taken into account. The findings of this study cannot be used as a representative of the underlying population as the patients are more likely to be enlightened than the general population about risk factors and prevention of CVD. The study was a cross sectional survey using questionnaires and medical records and therefore no causal relationships could be precisely defined as the patients were not followed up. Despite these limitations, the findings of the study can be generalized to the patients attending the diabetes and hypertension management clinics in these two slum settings. The study is unique as it is the first to investigate the awareness of risk factors for CVD among diabetic/hypertensive patients in the slums of Kenya. In addition, proper diagnosis of diabetes and/or hypertension had been done as respondents being interviewed were not attending the clinics for the first time.

## Conclusion

Risk factors for CVD remain prevalent among the patients attending diabetes and hypertension management clinics in the two Nairobi slums. The presence of CVD among the patients was influenced by gender and poor awareness of the link between hypertension and CVD. Understanding the etiology, natural history, and management paradigms of the CVD risk factors among the diabetic/hypertensive patients is therefore crucial in risk factor management so as to prevent or delay the development of CVD. Results from this study can be used in the establishment of CVD risk factor awareness and health educational programmes within the diabetes and hypertension management clinics in similar settings. These can then be adopted by the diabetic/hypertensive patients attending the outreach clinics to complement other preventive measures as they are more likely to develop CVD compared to the general population.
